# Penetration of capecitabine and its metabolites into malignant and healthy tissues of patients with advanced breast cancer

**DOI:** 10.1038/sj.bjc.6600809

**Published:** 2003-03-04

**Authors:** R M Mader, C Schrolnberger, B Rizovski, M Brunner, C Wenzel, G Locker, H G Eichler, M Mueller, G G Steger

**Affiliations:** 1 Clinical Division of Oncology, Department of Medicine I, University Hospital, Waehringer Guertel 18-20, A-1090 Vienna, Austria; 2 Division of Clinical Pharmacokinetics, Department of Clinical Pharmacology, University Hospital, Waehringer Guertel 18-20, A-1090 Vienna, Austria

**Keywords:** capecitabine, microdialysis, pharmacokinetics, tissue exposure, metabolism

## Abstract

Capecitabine is an oral prodrug of 5-fluorouracil (FU). Since FU concentrations achieved in malignant lesions are an important determinant of efficacy, we investigated the intratumoral transcapillary transfer of capecitabine and its metabolites *in vivo*. A total of 10 patients with skin metastases from breast cancer received a daily dose of 2500 mg m^−2^ capecitabine administered orally in two divided doses for 2 weeks. Microdialysis probes were inserted into a cutaneous metastasis and subcutaneous connective tissue to evaluate the interstitial tissue pharmacokinetics of capecitabine and its metabolites 5′-deoxy-5-fluorocytidine (DFCR), 5′-deoxy-5-fluorouridine (DFUR), and FU by capillary electrophoresis. As intended with the prodrug design of capecitabine, FU was present in low concentrations in tumour interstitium (median *c*_max_: 0.26 *μ*g ml^−1^) when compared with capecitabine, DFCR, and DFUR (median *c*_max_: 2.66, 4.22, and 2.13 *μ*g ml^−1^, respectively). Capecitabine and its metabolites easily penetrated malignant and healthy tissue and equilibrated within 45 min between plasma and tissue interstitium. Considering tissue exposure at the extracellular level, no significant differences between healthy and malignant tissues were observed. Our data show that absorption and metabolism determined the tissue pharmacokinetics of capecitabine. There was no evidence of drug tolerance, which may be attributed to impaired transcapillary transfer into tissue, even after repeated administration as shown for three patients.

Capecitabine is an oral prodrug of 5-fluorouracil (FU), which is activated mainly in liver and tumour via a cascade of three enzymes (Miwa *et al*, 1998). Exploiting this enzymatic cascade, capecitabine has been rationally designed to achieve a tumour selective accumulation of FU. This is finally carried out by the enzyme thymidine phosphorylase, which is often highly expressed in malignant tissue. Once built up, FU is then anabolysed intracellularly to its cytotoxic species. In contrast, the catabolic sequence of FU is initiated by the enzyme dihydropyrimidine dehydrogenase and degrades FU to compounds lacking antiproliferative activity. The pharmacokinetics of capecitabine and its metabolites have been described in detail when given either as monotherapy (Budman *et al*, 1998; Mackean *et al*, 1998; Judson *et al*, 1999) or after biochemical modulation with folinic acid (Cassidy *et al*, 1998). In early clinical trials, capecitabine has been used in combination with paclitaxel (Villalona-Calero *et al*, 1999) or with docetaxel (Pronk *et al*, 2000). Both taxanes did not affect the pharmacokinetics of capecitabine, although upregulation of thymidine phosphorylase has been reported in tumour xenografts (Ishitsuka, 2000) after exposure to paclitaxel or docetaxel. Mild to moderate hepatic dysfunction had no significant influence on the pharmacokinetics of capecitabine and its metabolites (Twelves *et al*, 1999), whereas food had a profound effect on the area under the concentration – time curve (AUC) of capecitabine (Reigner *et al*, 1998).

In contrast to the well-described pharmacokinetics in blood, data about intratumoral pharmacokinetics are scarce. In nude mice, the enrichment of FU in tumour xenografts was remarkably high (Ishikawa *et al*, 1998). In biopsies of human colon tumours, the accumulation of FU was less favourable when compared with the mouse model. Using tissue biopsies obtained sporadically during routine surgery, this investigation has shown the selective activation of capecitabine to FU in primary colorectal tumours, but not in liver metastases when compared with surrounding healthy liver tissue (Schüller *et al*, 2000).

To gain insight into tissue pharmacokinetics, we chose the microdialysis technique as an approach for the continuous monitoring of capecitabine and its metabolites in the extracellular extravascular space. In the present investigation, we compare the pharmacokinetics of capecitabine and its metabolites in blood, malignant and healthy tissue interstitium in the form of the free, nonprotein-bound drug. The aim of this study was to assess the extravascular transfer of capecitabine and its metabolites into healthy and malignant tissues and to characterise putative differential tissues distribution as a starting point to unravel the mechanism of unwanted side effects of this drug.

## MATERIALS AND METHODS

The clinical study protocol was approved by the local ethics committee. All patients were given a detailed description of the study and their written informed consent was obtained. The study was performed in accordance with the declaration of Helsinki and the Good Clinical Practice Guideline of the European Commission.

### Patient selection

Patients with a histologically confirmed diagnosis of breast cancer with skin metastases to the chest wall suitable for the insertion of microdialysis probes were admitted to the study. These patients were resistant to both paclitaxel and anthracycline containing chemotherapy regimens or had previously received cumulative doses of 400 mg m^−2^ of doxorubicin or doxorubicin equivalents. Women of childbearing potential were advised to avoid becoming pregnant while receiving capecitabine treatment. A pregnancy test was performed prior to the study day to exclude a present pregnancy. Patients older than 19 years with a WHO performance status (ECOG scale) 0, 1, or 2 were eligible. Criteria for inclusion were as follows: histologic proof of breast cancer, age 19–80 years, Karnofsky performance status >80%, absence of distant disease, adequate haematologic parameters (white blood count ⩾3.500 *μ*l^−1^, haemoglobin level >9 g dl^−1^, and platelet count ⩾100.000 *μ*l^−1^), adequate hepatic (serum bilirubin <1.5 mg dl^−1^, transaminases < twice the upper limit of normal) and renal functions (serum creatinine <1.5 mg dl^−1^).

Patients with a pathologic coagulation time presenting a risk for probe insertion, that is, a PTT >41.0 s or PTT <75%, and a known hypersensitivity to FU were excluded from the study.

### Treatment plan

The daily dose of capecitabine was 2500 mg m^−2^ administered orally with food according to the manufacturer's instructions for 2 consecutive weeks followed by a 1-week rest period (=1 cycle). The daily dose was given orally in two divided doses (approximately 12 h apart) within 30 min after a meal. Tablets (Xeloda®, Roche, Basel, Switzerland) were swallowed with tap water.

### Microdialysis

Patients were in a supine position throughout the study period. Commercially available microdialysis probes (CMA 10®, CMA, Stockholm, Sweden) were inserted into a suitable cutaneous metastasis. A second microdialysis probe was inserted horizontally into the subcutaneous connective tissue of one thigh. The surface of the disinfected skin was punctured according to a previously described procedure (Müller *et al*, 1995). The position of the probes in the metastasis was established by ultrasound scanning. Subsequently, the microdialysis system was connected and perfused with Ringer's solution at a flow rate of 1.5 *μ*l min^−1^ by a microinfusion-pump (Precidor®; Infors-AG, Basel, Switzerland). After a 30 min baseline sampling period, probe calibration for capecitabine and FU was performed for a period of 30 min according to a retrodialysis procedure (Stahle *et al*, 1991). After a wash-out period of 30 min, the study drug was administered in the scheduled dose. Sampling was continued at 30-min intervals up to 300 min. This interval corresponds to four plasma half-lives of the metabolite DFCR (half-life: 1.22 h) as the most slowly eliminating compound (Mackean *et al*, 1998).

As a prerequisite for this study, several quality control parameters were assessed using an *in vitro* microdialysis system. The recovery was absolutely constant over 2 h for capecitabine, DFCR, DFUR, and FU. For these compounds, the recovery was independent of the concentration up to 25 *μ*g ml^−1^. The lowest recovery was observed for the most lipophilic compound capecitabine (51.8%) when compared with the most hydrophilic substance FU (72.9%) with intermediate recoveries for the nucleosides DFCR and DFUR (60.7 and 61.8%, respectively). Since the *in vivo* recovery of DFCR and DFUR could not be evaluated and considering that both metabolites behave similar to FU (no plasma protein binding as shown below and *in vitro* recoveries similar to FU), DFCR and DFUR were calculated using the *in vivo* recovery of FU.

In parallel, a plastic cannula was inserted into an antecubital vein to monitor blood concentrations of the study drug at 30-min intervals. A total of 10 capecitabine-naive patients were studied after the first morning dose of day 1. Three of them were monitored once again between days 12 and 14 of cycle 1.

### Analysis of samples

Sample collection and analysis of microdialysates and blood samples by capillary electrophoresis were performed according to a validated method with minor modifications (Mader *et al*, 1998). After thawing, 100 *μ*l plasma were centrifuged through Centrisart-C4 filter units with a nominal molecular weight cutoff=10 000 (Sartorius, Germany) for 30 min at 37°C to separate free drug from protein-bound drug. After ultrafiltration, the analytical results obtained from blood samples were directly compared with those obtained from microdialysates, which contained only free drug. Capecitabine, DFCR, DFUR (supplied by Roche, Basel, Switzerland), and FU (Sigma, St Louis, MO, USA) were evaluated in 200 mM tetraborate buffer (pH=9.5) monitoring the absorbance at three different wavelengths (capecitabine: 297 nm, DFCR: 282 nm, DFUR and FU: 268 nm). Typical migration times were 10.4 min for DFCR, 12.9 min for capecitabine, 17.8 min for FU, and 19.3 min for DFUR. The detection limit was 0.1 *μ*g ml^−1^ for all evaluated compounds. The intra-assay coefficient of variation for this method ranged from 3.4 to 6.9% with an interassay coefficient of variation ranging from 6.7 to 10.4%.

The plasma protein binding of DFCR, DFUR, and FU was <4% up to 100 *μ*g substance (ml plasma)^−1^. In contrast, the plasma protein binding of capecitabine was determined to be 60% by *in vitro* binding experiments (data not shown).

### Data analysis

To obtain absolute interstitial concentrations from dialysate concentrations, microdialysis probes were calibrated for *in vivo* recovery rates according to the retrodialysis method (Müller *et al*, 1995,^1997^). The principle of this method relies on the assumption that the diffusion process is quantitatively equal in both directions across the semipermeable membrane. Therefore, the study drug is added to the perfusate and the disappearance rate through the membrane is taken as the *in vivo* recovery. The *in vivo* recovery value is calculated as:


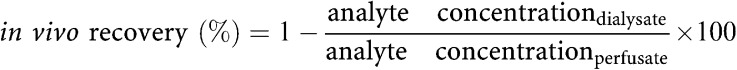


In the present study, the recovery of capecitabine was similar to that of FU in both tissues (range_capecitabine_ in tumour: 11–58%, range_FU_ in tumour: 13–75%, range_capecitabine_ in subcutaneous connective tissue: 13–65%, range_FU_ in subcutaneous connective tissue: 12–69%).

Absolute interstitial fluid concentrations of free drug were calculated from dialysate concentrations by the following equation:


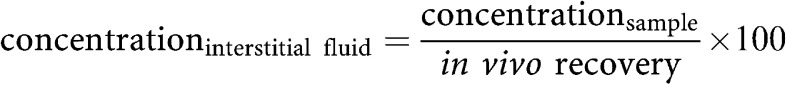


To avoid interferences with the subsequent pharmacokinetic investigation, a very low concentration of capecitabine and FU was used for the retrodialysis *in vivo* (2 *μ*g ml^−1^). The very small amount of drug transferred into tissue during the retrodialysis (90 ng of each substance using a flow rate of 1.5 *μ*l min^−1^ over 30 min) resulted in blank electropherograms after a wash-out period of 30 min.

The following pharmacokinetic parameters were determined: AUC calculated using the trapezoidal rule, maximum concentration (*c*_max_), time to maximum concentration (*t*_max_), and elimination half-life (*t*_1/2_). For statistical calculations, pharmacokinetic parameters derived from different compartments were compared using the paired Wilcoxon test.

## RESULTS

After peroral administration to capecitabine-naive patients, the pharmacokinetics of capecitabine varied considerably from patient to patient when considering the systemic circulation (capecitabine in plasma, range of *c*_max_: 0.64–15.4 *μ*g ml^−1^, range of *t*_max_: 1.0–3.0 h). Common features of the plasma pharmacokinetics included the dominance of metabolism in the form of the FU precursors DFCR and DFUR (Table 1

**Table 1:** Pharmacokinetic parameters^a^ of capecitabine and its metabolites in plasma, malignant tissue, and healthy connective tissue

**Compartment**	**Parameter**	**Capecitabine**	**DFCR**	**DFUR**	**FU**
Plasma	*c*_max_ (*μ*g ml^−1^)	2.34	6.53	3.65	0.25
		(0.64–15.4)	(1.79–8.7)	(1.67–7.22)	(0.09–0.80)
	*t*_max_ (h)	1.75	1.75	2.0	2.5
		(1.0–3.0)	(1.0–3.0)	(1.0–3.0)	(1.0–3.5)
	*T*_1/2_ (h)	0.50	0.86	0.80	N.A.
		(0.21–0.92)	(0.6–2.33)	(0.44–2.44)	
	AUC_0−5 h_ (*μ*g ml^−1^ h)	2.36	11.3	6.42	0.29
		(1.13–17.3)	(4.74–16.5)	(4.26–10.0)	(0.13–1.86)
					
Tumour	*c*_max_ (*μ*g ml^−1^)	2.66	4.22	2.13	0.26
		(0.91–15.7)	(1.54–4.29)	(0.85–4.29)	(0–0.74)
	*t*_max_ (h)	2.5	2.5	2.5	3.0
		(1.5–3.5)	(1.5–3.5)	(1.5–4.0)	(1.0–4.5)
	*t*_1/2_ (h)	0.50	0.87	1.08	N.A.
		(0.27–1.24)	(0.43–1.59)	(0.48–2.17)	
	AUC_0−5 h_ (*μ*g ml^ 1^ h)	3.52	7.89	3.35	0.25
		(0.92–33.3)	(2.15–25.2)	(1.43–13.4)	(0–2.28)
					
s.c. Tissue	*c*_max_ (*μ*g ml^−1^)	2.70	4.37	1.51	0.33
		(0.58–28.2)	(1.11–10.1)	(0.70–5.87)	(0.08–1.40)
	*t*_max_ (h)	2.25	2.5	2.75	2.0
		(1.0–3.5)	(1.5–3.5)	(1.5–3.5)	(0.5–3.5)
	*t*_1/2_ (h)	0.59	0.76	0.82	N.A.
		(0.17–1.5)	(0.57–3.57)	(0.64–2.80)	
	AUC_0−5 h_ (*μ*g ml^−1^ h)	3.16	8.40	2.75	0.36
		(0.78–43.5)	(2.31–22.1)	(1.51–11.6)	(0.13–2.21)
					

**Figure 1:**
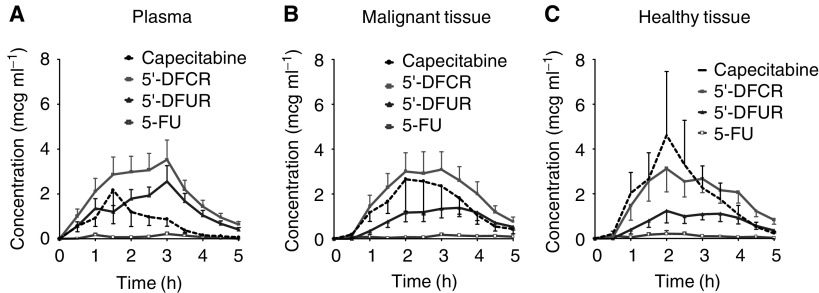
Concentration–time profiles of capecitabine and its metabolites in patients with advanced breast cancer. Patients received 1250 mg capecitabine m^−2^ orally with tap water before collecting plasma samples and interstitial tissue fluid up to 5 h after administration. Concentration–time profile in plasma (**A**), concentration–time profile in the interstitium of malignant tissue (**B**), concentration–time profile in the interstitium of subcutaneous connective tissue (**C**). Concentrations are depicted as means of the single courses ± s.e. (*n*=10). 5′-DFCR=5′-deoxy-5-fluorocytidine; 5′-DFUR=5′-deoxy-5-fluorouridine; 5-FU=5-fluorouracil.

Patients received 1250 mg capecitabine m^−2^ orally with tap water before collecting plasma samples and interstitial tissue fluid up to 5 h after administration.

a Values are reported as median and range in parentheses (*n*=10). DFCR=5′-deoxy-5-fluorocytidine; DFUR=5′-deoxy-5-fluorouridine; FU=5-fluorouracil; *c*max=maximum concentration; *t*max=time to maximum concentration; *t*_1/2_=elimination half-life; AUC_0−5 h_=area under the concentration-time curve from 0 to 5 h; NA=not applicable.

). In contrast to FU, plasma concentrations observed after i.v. infusion, this regimen was characterised by low FU levels rarely exceeding 0.5 *μ*g ml^−1^ plasma (Figure 1A

).

Considering distribution processes, capecitabine and its metabolites DFCR and DFUR easily penetrated malignant and healthy tissues (Figure 1B and C). Equilibration between plasma and tissue interstitium occurred within 45 min as determined from the time interval between the peak concentrations in plasma and tissue (Table 1).

In the malignant lesion, the exposure to capecitabine was significantly higher when compared with plasma (AUC in tumour *vs* plasma: *P*=0.01), whereas the AUC of all other metabolites did not differ significantly between malignoma and plasma. Comparing the interstitium of subcutaneous connective tissue with plasma, only the AUC of DFUR was significantly lower in healthy tissue (*P*=0.014). There was, however, a trend for higher exposure to capecitabine in subcutaneous tissue in a subgroup of four patients (ratio AUC_connective tissue_/AUC_plasma_>2).

FU observed in the systemic circulation equilibrated rapidly into interstitial fluid without preference for malignant or healthy tissue. Considering tissue exposure of all evaluated compounds (in terms of AUC), no statistically significant differences were observed in the interstitial space of malignant lesions and subcutaneous connective tissue.

Since there is modest enzymatic activation of capecitabine already in the stomach followed by rapid distribution from blood to tissue, we questioned the tissue-specific transfer among these compounds as expressed in the ratio AUC tissue/AUC plasma (Table 2

**Table 2:** Transcapillary transfer^a^ of capecitabine and its metabolites to malignant and healthy tissues

**Ratio of AUC_0−5 h_**	**Capecitabine**	**DFCR**	**DFUR**	**FU**
Tumour/plasma	1.76	0.67	0.53	0.66
	(0.54–7.07)	(0.45–2.97)	(0.28–2.56)	(0–5.56)
s.c. Tissue/plasma	1.31	0.67	0.44	1.53
	(0.46–16.7)	(0.41–2.60)	(0.25–1.36)	(0.32–3.38)
Tumour/s.c. tissue	1.11	1.12	1.20	0.66
	(0.36–1.99)	(0.55–1.93)	(0.60–3.07)	(0–17.5)

a Ratios are reported as median and range in parentheses as calculated from each single patient (*n*=10). See Table 1.

). Capecitabine showed significantly higher differential transfer to the metastatic lesion when compared with the metabolites DFCR (ratio AUC tumour/AUC plasma of capecitabine *vs* DFCR: *P*=0.004) and DFUR (*P*=0.002). A similar observation was made for subcutaneous connective tissue (ratio AUC connective tissue/AUC plasma of capecitabine *vs* DFCR: *P*=0.002; ratio AUC connective tissue/AUC plasma of capecitabine *vs* DFUR: *P*=0.002). Considering the ratio AUC tumour/AUC connective tissue for all evaluated compounds, there was no statistically significant difference between both tissues. When considering the maximum concentration, however, the ratio *c*_max_ tumour/*c*_max_ connective tissue of FU was significantly lower than that of all other evaluated compounds (ratio *c*_max_ tumour/*c*_max_ connective tissue of capecitabine *vs* FU: *P*=0.05; DFCR *vs* FU: *P*=0.03; DFUR *vs* FU: *P*=0.05). Besides statistical calculations, FU peak concentrations in connective tissue exceeded twice that of malignant tissue in six of 10 capecitabine-naive patients. Given the constantly low concentrations of FU in plasma and in interstitial tissue fluid, no correlation between pharmacodynamics and tissue FU kinetics could be established. The highest haematologic toxicity was observed in one patient with anaemia WHO grade 2 and four patients suffering from anaemia WHO grade 2.

**Figure 2:**
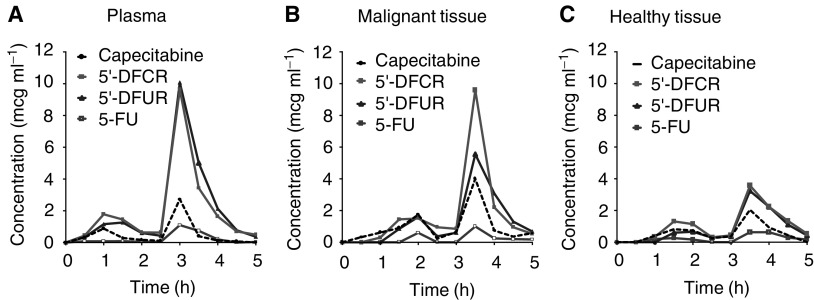
Concentration–time profiles of capecitabine and its metabolites in one patient on day 12. Patients received 1250 mg capecitabine m^−2^ orally with tap water before collecting plasma samples and interstitial tissue fluid up to 5 h after administration. Concentration–time profile in plasma (**A**), concentration–time profile in the interstitium of malignant tissue (**B**), concentration–time profile in the interstitium of subcutaneous connective tissue (**C**).

To consider repeated administration of capecitabine, drug monitoring was repeated at the end of the first therapeutic cycle in three patients (days 12–14). In one of these patients, absorption and distribution were subject to remarkable variations between different drug applications. Nevertheless, this patient illustrates a close relation between plasma kinetics and interstitial tissue kinetics observed throughout this study. When capecitabine was administered on day 1, the metabolites DFCR and DFUR did not exceed plasma concentrations of 4 *μ*g ml^−1^ in one patient because of slow absorption kinetics without pronounced absorption maxima. Although FU was detected in plasma and in the connective tissue of this patient, FU was not observed in the tumour interstitium. When given at the end of the first cycle, absorption started later in comparison to day 1, but the plasma concentrations of the metabolites DFCR and DFUR were about 10 *μ*g ml^−1^ with clearly defined absorption maxima. In addition, the distribution into subcutaneous connective tissue was high on day 1, but was low at the end of the first cycle (Figure 2A–C

). During this administration, the concentrations and the AUC of FU were very similar in plasma and in tissue interstitium. In the other patients, the differences from course to course were in the range expected after peroral administration as estimated by the ratio of AUC and *c*_max_ (differences between the courses in general <35%).

## DISCUSSION

The peroral formulation of capecitabine has spurred new expectations exploiting the efficiency of the well-known antimetabolite FU in cancer chemotherapy. For the time being, the description of the systemic pharmacokinetics of capecitabine is only matched sporadically by pharmacokinetic data derived from human tumours *in vivo*. Besides tumour xenografts, there is evidence of a selective enrichment of FU in colorectal tumours. Considering the concentration of FU in tissue biopsies, the median ratio tumour/healthy tissue was 2.9 with a median tumour/plasma ratio of 16.6. In secondary lesions, no selectivity towards liver metastases *vs* healthy liver tissue was observed (Schüller *et al*, 2000). Limitations of biopsies, however, are the lack of discrimination among different organ compartments, that is, the intracellular space and the interstitial fluid, and the sporadic sampling frequency. To gain further insight into the tissue pharmacokinetics of capecitabine using continuous monitoring, the microdialysis technique was employed in the present investigation.

After absorption, the distribution of capecitabine and its metabolites occurred within 45 min into tissue with little discrimination for malignant and subcutaneous connective tissues. The data shown in Figure 2 emphasise the absorption of capecitabine as a decisive step for the interstitial tissue kinetics of capecitabine and its metabolites. As a consequence, blood peak levels of the monitored compounds were similar to those in tissue interstitium. With exception of the parent drug capecitabine, the complete equilibration between plasma and tissue interstitium precluded significant accumulation of the metabolites. Thus, the carboxylic side chain of capecitabine is believed to be responsible for the enhanced tissue affinity of the parent drug, which is then lost after conversion to its metabolites.

The efficacy of capecitabine relies on the cytotoxic mechanisms of FU. Once entered the cell, the balance between anabolic conversion to the cytotoxic species and catabolic degradation determines the pharmacodynamic activity. We have previously shown that the extravascular transfer of FU may be a critical parameter in determining the clinical response (Müller *et al*, 1997). After i.v. administration of FU, its transfer into primary breast cancer lesions was impaired in nonresponders. In the present investigation, the rapid and quantitative equilibration of capecitabine and its metabolites between plasma and tissue interstitium left little space for resistance to capecitabine caused by impaired tissue transfer of the parent drug or its metabolites.

Schüller *et al* (2000) have shown that at least in the colon and in the liver, the intracellular catabolism of FU via dihydropyrimidine dehydrogenase is similar in healthy and malignant tissues. As a consequence, it is reasonable to assume that the expected toxicity in healthy tissue will largely depend on the last activation step, which is the intracellular conversion of DFUR to FU via thymidine phosphorylase. In this context, the differential affinity of FU to subcutaneous connective tissue when compared with malignant tissue may offer a starting point to unravel the mechanism underlying the hand–foot syndrome, which may restrict therapeutic interventions with capecitabine. Although there was no statistically significant difference between FU pharmacokinetics in connective and malignant tissues, FU peak concentrations indicated an increased transfer into connective tissue in six of 10 patients. None of these patients, however, suffered from a hand–foot syndrome suggesting the additional effect of factors beyond FU as mechanism for this side effect. Although the activity of this enzyme was not the aim of the present investigation, one may hypothesise that the higher FU levels in the connective tissue of more than half of the patients did not result in toxic side effects because of lacking activation of DFUR to FU via thymidine phosphorylase. This is compatible with several previous observations about the pivotal role of this enzyme in the antiproliferative effect of FU (Schwartz *et al*, 1995; Ciccolini *et al*, 2001) and DFUR (Evrard *et al*, 1999a). Moreover, our data suggest that once converted to FU intracellularly, this agent is hardly redistributed to the systemic circulation thus contributing to the mild systemic side effects of large doses of capecitabine.

Surprisingly, there was little selectivity for the evaluated compounds when comparing the interstitium of malignant and healthy tissues. This may be partly because of the specific nature of skin metastases in terms of vascularisation and/or differential thymidine phosphorylase activity, which varies considerably among various tissues (Ackland and Peters, 1999). Given that the microdialysis technique allows for the monitoring of the extracellular space only, intracellular tissue selective activation can only be monitored after efflux of the metabolite from the cell. Therefore, intracellularly generated nucleosides and nucleotides such as fluorodeoxyuridine monophosphate, fluorodeoxyuridine triphosphate, and fluorouridine triphosphate cannot be directly assessed using this approach, because they are trapped within the cell after phosphorylation.

In the tumour, the activity of thymidine phosphorylase may be critical, because this enzyme plays a double role in the activation of capecitabine. Depending on the cellular concentration, thymidine phosphorylase is able to convert DFUR to FU intracellularly, but also to further activate FU to fluorodeoxyuridine. Only then, the cytotoxic potency of FU is fully exploited. Unfortunately, the regulation of this enzyme is not completely understood. *In vitro*, the expression of thymidine phosphorylase was subject to little variation after exposure of naive colon tumour cells to FU (Mader *et al*, 1997). Its importance in the conversion of FU, however, has been definitely confirmed (Evrard *et al*, 1999a,1999b). Thus, in addition to impaired absorption of capecitabine, the activity of thymidine phosphorylase is very likely to be a critical factor in the therapy with capecitabine.

For an agent administered daily, one of the most important clinical questions considers possible alterations of pharmacologic parameters with time. As shown for the small number of three patients, the repeated administration did not alter the tissue transfer or the metabolism of the drug significantly. Since at least two of the enzymes involved in the activation of capecitabine, that is, carboxylesterase and cytidine deaminase, may be monitored via the rate of the formation of the metabolites DFCR and DFUR in plasma, one may assume a stable metabolic situation within one therapeutic cycle (28 administrations) with a daily dose of 2500 mg capecitabine m^−2^. Although the limited number of observations precludes definite conclusions, these data suggest the absence of pharmacokinetic mechanisms related to capecitabine resistance.

Since patients did previously receive anthracycline- or taxane-based regimens, one possible mechanism of resistance should consider upregulation of MDR1/P-glycoprotein. In this study, the hydrophilic metabolites DFCR and DFUR were mainly observed in tissue interstitium. Although upregulation of MDR1/P-glycoprotein may be observed for a median period of 4 months after therapy with modulating agents such as anthracyclines or taxanes (M Filipits, personal communication), there is no evidence in the literature that the cellular uptake of FU and fluoropyrimidine nucleosides is influenced by the expression of MDR1/P-glycoprotein.

Although one restriction of the microdialysis technique is the inability to monitor intracellular processes, this technique allows for the evaluation of drugs in a well-defined tissue compartment, namely the extravascular interstitial tissue fluid. This is in contrast to all other analytical techniques applied to fluoropyrimidine pharmacokinetics such as tumour biopsies or NMR-studies, which concomitantly monitor signals in intra- and extracellular compartments.

The results from this study show that capecitabine and its metabolites DFCR and DFUR distribute extensively into the interstitium of malignant and healthy tissues. This efficient penetration into the interstitium of malignant tissue is the initial necessary step before DFUR enters tumour cells, where it is converted to FU by thymidine phosphorylase. The results obtained in the present study are complementary to those obtained previously by Schüller *et al* (2000), who reported a mean tumour/plasma ratio of 21 for FU in patients with colorectal tumours. This very high concentration of FU in malignant tissue compared to plasma could only be achieved because, as a first step, the precursors of FU distributed very efficiently into the interstitium of malignant tissue, as demonstrated in the present study.

In conclusion, capecitabine and its metabolites DFCR and DFUR easily penetrated malignant and healthy tissues with little selectivity among both types of tissues. Concomitantly, the concentrations of FU were low in blood and in the interstitium of malignant and healthy tissues. Our data show that absorption and metabolism determined the tissue pharmacokinetics of capecitabine. There was no evidence of drug tolerance, which may be attributed to impaired transcapillary transfer into tissue, even after repeated administration of capecitabine as shown for three patients.
